# Could whole-body cryotherapy (below −100°C) improve muscle recovery from muscle damage?

**DOI:** 10.3389/fphys.2014.00247

**Published:** 2014-07-02

**Authors:** Joao B. Ferreira-Junior, Martim Bottaro, Jeremy P. Loenneke, Amilton Vieira, Carlos A. Vieira, Michael G. Bemben

**Affiliations:** ^1^College of Physical Education, University of BrasíliaBrasília, Brazil; ^2^Federal Institute of Triângulo MineiroParacatu, Brazil; ^3^Neuromuscular Research Laboratory, Department of Health and Exercise Science, The University of OklahomaNorman, OK, USA

**Keywords:** muscle function, inflammation, oxidative stress, recovery modalities, adhesion molecules

Muscle performance might be temporarily impaired by high-intensity exercise performed during a competition or training session. The attenuation in muscular strength may be transitory, lasting minutes, hours, or several days following training or competition (Barnett, 2006). Longer-lasting impairment in muscle strength accompanied by a reduction in range of motion, an increase in muscle proteins in the blood, an inflammatory response, muscle swelling, and delayed onset muscle soreness is referred to as exercise induced muscle damage (EIMD) (Clarkson and Hubal, 2002; Barnett, 2006; Paulsen et al., 2012).

Different modalities have been used to improve recovery from a damaging bout of exercise (Barnett, 2006). Among the most common treatment approaches used to reestablish muscular function are active recovery, compression garments, massage, stretching, anti-inflammatory drugs, and cryotherapy (Cheung et al., 2003; Barnett, 2006; Bishop et al., 2008). A relatively novel modality of cryotherapy is whole-body cryotherapy (WBC), which consists of brief exposure (2–3 min) to extremely cold air (−100 to −195°C) in a temperature-controlled chamber or cryocabin (Banfi et al., 2010; Hausswirth et al., 2011; Fonda and Sarabon, 2013). Sessions of partial-body cryotherapy (PBC), in which the head is not exposed to cold, has also been used as a similar modality of WBC (Hausswirth et al., 2013). According to Hausswirth et al. (2013), WBC and PBC session decreased skin temperature, however, WBC induced a greater decrease compared to PBC. In addition, the tympanic temperature was reduced only after the WBC session. Moreover, parasympathetic tone stimulation was greater following the WBC session. Although WBC has been used since the end of the 1970s in the treatment of rheumatic diseases (Ksiezopolska-Pietrzak, 2000; Metzger et al., 2000; Rymaszewska et al., 2003), it has only recently been used with the purpose of hastening recovery from muscle damage by decreasing the inflammatory process linked to EIMD (Banfi et al., 2010). A logic model proposed by Costello et al. (2013) consisted of the physiological, neuromuscular, and perceptual effects following exposure to WBC which may interact to increase performance. However, a mechanistic model for how WBC may improve symptoms related to EIMD has to this point not been provided (Costello et al., 2013). Thus, the purpose of this manuscript was to briefly address a possible mechanism related to improved recovery from muscle damage by WBC.

## Muscle damage induced by exercise

The “popping-sarcomere” hypothesis, first proposed by Morgan in (1990), provided an explanation for the muscle damage response following a series of eccentric contractions. Since then, this “popping-sarcomere” hypothesis has garnered support from several other authors (Clarkson and Hubal, 2002; Peake et al., 2005; Paulsen et al., 2012). It's hypothesized that during an eccentric contraction, myofibrils within a muscle fiber are stretched and those weaker sarcomeres absorb most of the stretch. Following several eccentric contractions, the myofilaments of overstretched weaker sarcomeres fail to reconnect because they are disrupted. This disruption may extend to other sarcomeres, resulting in cell membrane and sarcoplasmic reticulum damage. This process appears responsible for the symptoms associated with EIMD, such as the prolonged loss in muscle strength, delayed-onset muscle soreness, increase of muscle protein in blood circulation, intracellular calcium release and onset of the inflammatory response (Clarkson and Hubal, 2002; Peake et al., 2005; Paulsen et al., 2012).

**Figure 1:**
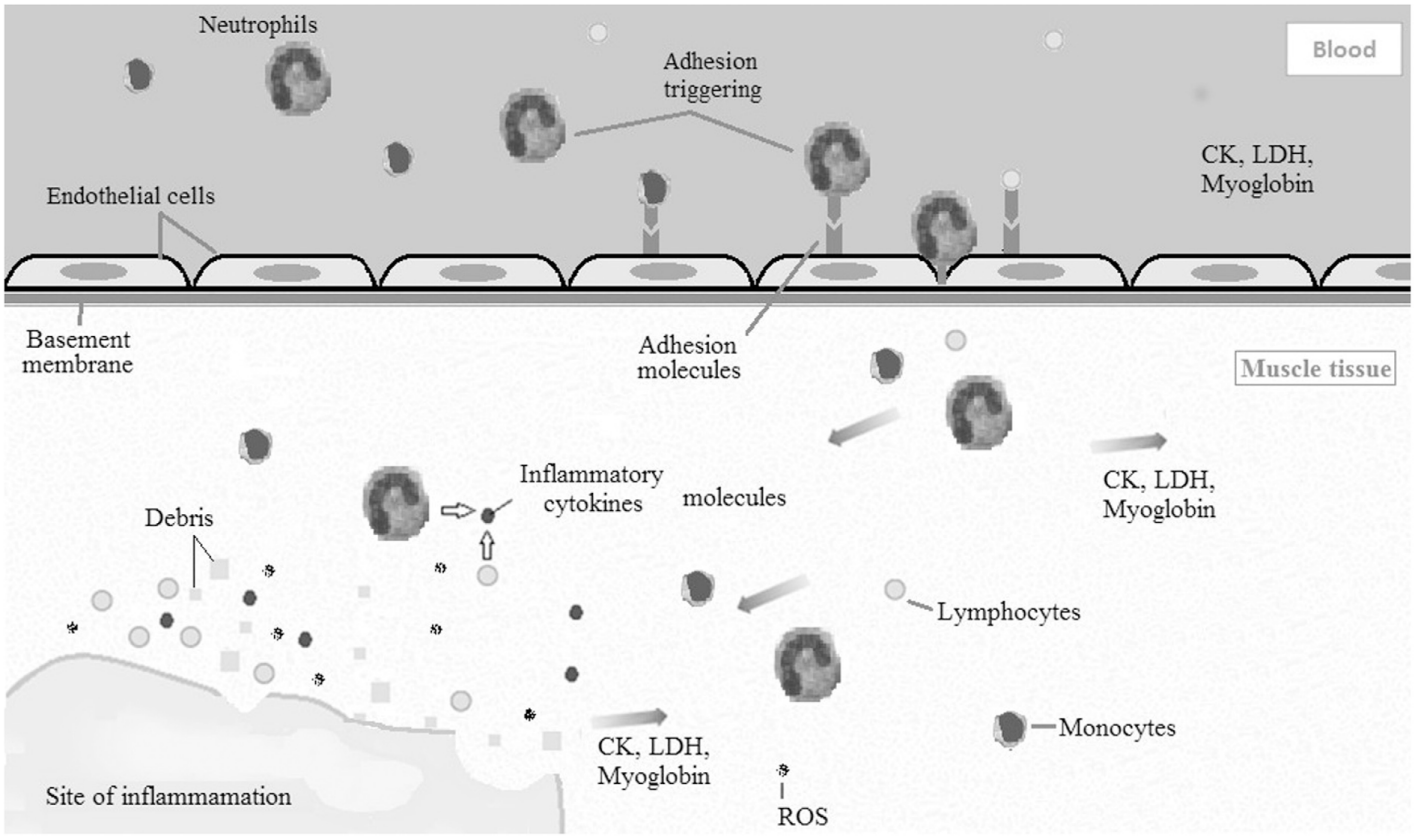
**Diagram illustrating the acute inflammatory process caused by exercise induced muscle damage (EIMD)**. Leukocytes (neutrophils, monocytes, and lymphocytes) are mobilized to the damaged muscle tissue via soluble intercellular adhesion molecule 1 (sICAM-1). Afterward, pro-inflammatory cytokines and reactive oxygen species are produced in muscle by leukocytes. Together, leukocytes, pro-inflammatory cytokines, and reactive oxygen species cause intramuscular degradation, which amplifies the initial muscle damage. CK, creatine kinase; LDH, lactate dehydrogenase; ROS, reactive oxygen species.

Immediately after sarcomeres are disrupted, leukocytes are mobilized to the injured tissue via soluble intercellular adhesion molecule 1 (sICAM-1). Thereafter, pro-inflammatory cytokines and reactive oxygen species are produced in muscle by neutrophils, lymphocytes, and monocytes (Clarkson and Hubal, 2002; Peake et al., 2005; Paulsen et al., 2012). Additionally, macrophages produced by monocytes and neutrophils phagocytize damaged myofibers and muscle debris (Peake et al., 2005; Saclier et al., 2013). Together (Figure 1), leukocytes, pro-inflammatory cytokines, and reactive oxygen species cause intramuscular degradation, which amplifies the initial muscle damage (Clarkson and Hubal, 2002; Peake et al., 2005; Paulsen et al., 2012). This can be observed by an increase in muscle proteins in systemic circulation 24–48 h following the initial bout of exercise. Additionally, the magnitude of the secondary muscle damage response may depend upon the balance between pro- and anti-inflammatory cytokines (Clarkson and Hubal, 2002).

## Previously reported mechanisms to WBC improves muscle damage recovery

To the best of our knowledge, four studies have evaluated the effects of WBC on the recovery from muscle damage (Hausswirth et al., 2011; Pournot et al., 2011; Fonda and Sarabon, 2013). Hausswirth et al. (2011) suggested that three sessions of WBC (3 min at −110°C) after EIMD in well-trained runners improved muscle strength, perceived sensation, and also decreased muscle pain. Additionally, five WBC exposures (3 min at −140 to −190°C) may improve the recovery of peak torque, rate of torque development, squat jump start power, and decreased muscle soreness after damaging exercise for the hamstrings (Fonda and Sarabon, 2013). Further, Pournot et al. (2011) found that three sessions of WBC (3 min at −110°C) following EIMD was effective in reducing the inflammatory response.

It is acknowledged that many of these reported effects of WBC on recovery from severe exercise are small (Bleakley et al., 2014). However, it is also acknowledged that those studies (Hausswirth et al., 2011; Pournot et al., 2011; Fonda and Sarabon, 2013) have been collected using a cross-over design and could have been influenced by the repeated bout effect. Preliminary data from our laboratory showed that a single WBC session performed immediately after EIMD relieved pain, prevented muscle swelling, and resulted in quicker recovery of muscle strength 72 h after EIMD. In contrast, the control group did not recover from muscle swelling and pain until 24 and 96 h following EIMD, respectively. Further, muscle strength in the control group was still depressed from baseline 96 h post exercise. In apparent contrast to our findings, Costello et al. (2012a) reported that one session of WBC (20 s at −60°C and 3 min at −110°C) applied 24 h after EIMD in healthy subjects did not hasten muscle strength nor decrease muscle soreness. However, the acute inflammatory process is triggered immediately following EIMD. Thus, it stands to reason that WBC applied 24 h after exercise may be too late to have a beneficial physiologic response. Therefore, WBC may decrease symptoms related to EIMD produced by mechanical stress if applied immediately after exercise (Hausswirth et al., 2011; Pournot et al., 2011; Fonda and Sarabon, 2013).

The aforementioned studies (Hausswirth et al., 2011; Pournot et al., 2011; Costello et al., 2012a; Fonda and Sarabon, 2013) were conducted with the assumption that WBC could accelerate the recovery from muscle damage by decreasing the inflammatory process. However, the mechanism behind this effect is largely unknown. Stanek et al. (2010) suggested that the anti-inflammatory effects of WBC may be linked to lysosomal membrane stabilization with a consequent inhibition of active enzymes released. Wozniak et al. (2007) showed a decrease in lysosomal enzyme activity: 32% in acid phosphatase and 46% in arylsulphatase after 6 days of WBC, but they were not altered after one session of WBC. Similarly, a single session of WBC did not cause a stabilization of lysosomal enzymes (Wozniak et al., 2007). Thus, considering the results of these studies (Dugue et al., 2005; Wozniak et al., 2007; Lubkowska et al., 2009), the hypothesis that lysosomal membrane stabilization is responsible for the anti-inflammatory effect of WBC might make sense after chronic exposure to WBC but not following an acute session.

## WBC improves muscle damage recovery by reducing sICAM-1?

We hypothesize that the thermoregulatory response to WBC may hasten the recovery from EIMD by reducing serum sICAM-1. The first step of this thesis is dependent upon the drop in core temperature, which would likely cause constriction of local arterioles and venules. In support of this, WBC exposure has been observed to result in a decrease in muscle and core temperature (Westerlund et al., 2003; Costello et al., 2012b). Costello et al. (2012b) observed a reduction of 0.3°C in rectal temperature 60 min after WBC and a similar decrease of 0.24°C was observed 20 min after WBC (Westerlund et al., 2003). It also has been reported that vastus lateralis temperature decreases 1.6 ± 0.6°C after WBC session (Costello et al., 2012b).

The next phase in this proposed mechanism is blocking the migration of leukocytes (neutrophils, lymphocytes, and monocytes) from blood circulation to the damaged tissue. It is known that leukocytes initiate the acute inflammatory process following EIMD (Clarkson and Hubal, 2002; Peake et al., 2005; Paulsen et al., 2012; Saclier et al., 2013). According to our hypothesis, the thermoregulatory response to WBC hastens the recovery from EIMD by reducing serum sICAM-1. Consequently, fewer neutrophils and lymphocytes would transmigrate into muscle tissue resulting in a decreased pro-inflammatory response (i.e., interleukin [IL]-2, IL-6, IL-8, IL-1β, prostaglandin [PGE]-2, and C-reactive protein), reactive oxygen species and an increased anti-inflammatory response (i.e., IL-10 and IL-1ra). The results reported by Pournot et al. (2011) and Mila-Kierzenkowska et al. (2013) corroborate this hypothesis. Pournot et al. (2011) observed an increase in IL-1ra and a decrease in IL-1β and C-reactive protein after the first session of WBC performed immediately following EIMD in well-trained runners. In addition, Mila-Kierzenkowska et al. (2013) found that a single session of WBC prior to 40 min of submaximal exercise decreased the level of IL-6, IL-1β, superoxide dismutase, and catalase activity when compared to exercise completed in the absence of WBC.

Further, it has been found that five sessions of WBC (30 s at 60°C and 2 min at −110°C) in athletes decreased adhesion molecule sICAM-I, blood concentrations of muscular enzymes (creatine kinase and lactate dehydrogenase), and the pro-inflammatory response (prostaglandin E2, interleukin IL-2, and IL-8) to 5 days of high intensity training (Banfi et al., 2009). In addition, the anti-inflammatory cytokine IL-10 was increased (Banfi et al., 2009). Nevertheless, these data should be interpreted with some degree of caution since this study did not evaluate a control group, and it evaluated chronic effect of WBC. Most studies investigating WBC and the inflammatory/oxidative response to exercise evaluated its effect following multiple sessions of WBC (Dugue et al., 2005; Wozniak et al., 2007, 2013; Leppaluoto et al., 2008; Banfi et al., 2009, 2010; Lubkowska et al., 2009, 2010, 2011, 2012; Mila-Kierzenkowska et al., 2009; Miller et al., 2012; Ziemann et al., 2012, 2013). Future research should investigate this further to determine if one application of WBC can in fact decrease sICAM-1. Nevertheless it stands to reason that WBC may attenuate symptoms of EMID by decreasing sICAM-1, subsequently reducing the migration of leukocytes into the damaged tissue.

## Conclusion

We wish to suggest that the attenuation in serum sICAM-1 caused by WBC exposure immediately following EIMD may be responsible for the decreased acute inflammatory response to muscle damage. In addition, repeated bouts of WBC may also further reduce the secondary inflammation occurring days after the damaging bout of exercise. However, the mechanisms in which the thermoregulatory effects of WBC exposure lead to a reduction in sICAM-1 remain unknown. Thus, further studies on this topic are necessary in order to better understand the thermoregulatory effects of WBC on muscle inflammatory process caused by EIMD. Future research could investigate this hypothesis assessing sICAM-1, oxidative stress, neutrophils, lymphocytes, monocytes, and cytokines cells following WBC applied immediately after EIMD. In addition, future work could investigate the effects of repeated WBC on long term muscle adaptation. Although potentially beneficial in the short term, it is unknown if suppressing the acute inflammatory response may negatively affect the muscles ability to adapt to exercise.

### Conflict of interest statement

The authors declare that the research was conducted in the absence of any commercial or financial relationships that could be construed as a potential conflict of interest.
